# Depression among people living with tuberculosis and tuberculosis/HIV coinfection in Ukraine: a cross-sectional study

**DOI:** 10.1080/16549716.2024.2448894

**Published:** 2025-02-13

**Authors:** Anna Salnikova, Olena Makarenko, Yuliia Sereda, Tetiana Kiriazova, Karsten Lunze, Jack DeHovitz, Danielle C. Ompad

**Affiliations:** a Ukrainian Institute on Public Health Policy, Kyiv, Ukraine; b Department of Medicine, Boston Medical Center, Boston, MA, USA; c Chobanian & Avedisian School of Medicine, Boston University, Boston, MA, USA; d Department of Medicine, SUNY Downstate Health Sciences University, Brooklyn, NY, USA; e Department of Epidemiology, New York University School of Global Public Health, New York, NY, USA; f Center for Drug Use and HIV, HCV Research, New York University School of Global Public Health, New York, NY, USA

**Keywords:** Depression, tuberculosis, HIV, mental health, Ukraine

## Abstract

**Background:**

Depressive disorders are associated with poor treatment outcomes, physical health, and quality of life among people living with TB (PLWTB) and TB/HIV (PLWTBHIV). Data on depression among PLWTB/HIV are limited in Ukraine.

**Objectives:**

This cross-sectional study aimed to examine depression risk and its correlates and describe the willingness to seek depression treatment among PLWTB/HIV in Ukraine.

**Methods:**

This secondary analysis included patients with and without HIV who initiated TB treatment within 30 days in two tertiary hospitals in Kyiv and Odesa. A survey was conducted from February 2021 to October 2022 and reviewed patients’ health records. We used the Center for Epidemiological Studies-Depression Scale (CES-D) to indicate risk for clinical depression. Factors associated with depressive symptoms were identified using logistic regression.

**Results:**

The sample included 209 participants (*n* = 100 with TB; *n* = 109 with TB/HIV). The mean age of participants was 43 (SD = 11) years; 66% of sample identified as male. Approximately 28% of participants were at risk for clinical depression; of whom 66% were willing to seek therapeutic or medical help. HIV coinfection (adjusted odds ratio [aOR] = 2.95, 95% confidence interval [CI]: 1.46,6.20), past 30 days illicit drug use (aOR = 3.57, 95% CI = 1.18,11.60), TB stigma (moderate stigma aOR = 7.40, 95% CI = 2.22,34.1; high stigma aOR = 15.50, 95% CI = 4.52,73.20), and unemployment status (aOR = 2.25, 95% CI = 1.12,4.60) were significantly associated with the odds of depressive symptoms among PLWTB.

**Conclusion:**

Findings support integration of a brief depression screening tool into routine clinical care of PLWTB/HIV and highlight the importance of linking TB/HIV care with mental health services.

## Paper Context

- **Main findings:** The prevalence of risk for clinical depression (28%) was substantial among people with TB and TB/HIV coinfection in Ukraine and a majority was willing to seek for therapeutic or medical help. HIV, illicit drug use, TB stigma, and unemployment contributed to the risk of depression among this population.
- **Added knowledge:** This analysis supports the importance of a comprehensive approach to mental health and HIV/TB services in Ukraine.
- **Global health impact for policy and action:** Projects aimed at integration and linkage development of mental health services and clinical care for TB/HIV, accessibility to comprehensive services for people with TB and TB/HIV coinfection could be supportive for their health and well-being.

## Background

Tuberculosis (TB) remains one of the major public health threats globally. In 2023, an estimated 10.8 million people were diagnosed with TB and a total of 1.55 million people died from TB [1]. Ukraine has one of the highest TB incidence rates in the World Health Organization (WHO) European Region and ranks among the countries with the highest burden of multidrug-resistant TB (MDR-TB) and rifampicin-resistant TB (RR-TB) [2]. Human immunodeficiency virus (HIV) coinfection accounted for approximately 20% of registered TB cases in Ukraine from 2015 to 2019 [3,4]. TB remains the main cause of death among adults living with HIV [5].

Depression is also a major public health burden and a leading cause of disability globally. The prevalence of depressive disorders in Ukraine was estimated to be 6.2% in 2019, which was higher than other countries in the region such as Poland (3.5%), Moldova (5.0%), and Estonia (6.0%) [6]. One in every eight Ukrainian adults reported symptoms consistent with clinical depression [7]; however, only one in four persons with potential depression had been informed by the healthcare professional or doctor that they had depression. In addition, only 0.4% of adults in Ukraine have been treated for depression with antidepressant medication or psychological therapy [7].

Depression is known to be associated with chronic diseases among adults [8–10], including TB. For people with TB and TB/HIV coinfection, depressive disorders have been associated with poor outcomes, including loss-to-follow-up, death during TB treatment, poor quality of life, and poor physical health [10–13]. The prevalence of depression is high among patients with TB [14]; however, data on depression and its correlates among patients with TB and TB/HIV is limited in Ukraine.

This study was conducted in the cities of Kyiv and Odesa, Ukrainian regions with high TB and HIV prevalence. For example, in 2019, TB prevalence in Ukraine was the highest in Odeska Oblast, with 150.3 cases per 100,000 population; 46.4% of whom were co-infected with HIV. In Kyiv, the proportion of TB/HIV coinfection among registered TB cases was the fourth highest (26.7%), exceeding the Ukrainian average (21.9%) [3]. The aim of the present study was to examine the risk for clinical depression and its correlates and describe willingness in treatment for depression among people living with TB (PLWTB) and TB/HIV (PLWTBHIV) in Ukraine.

## Methods

### Study setting

The parent cohort study was conducted by the Ukrainian Institute on Public Health Policy (UIPHP) and aimed to examine unhealthy alcohol use and other comorbidities among TB and TB/HIV co-infected patients in Ukraine. Participants were recruited from two tertiary hospitals in Kyiv and Odesa cities: Kyiv City Tuberculosis Hospital No 1 and Odesa Regional Center for Socially Significant Diseases. Participants were surveyed at baseline and 3 months post-baseline.

### Study design and duration

This analysis was a cross-sectional secondary data analysis of the baseline data that aimed to examine risk for depression among persons with diagnosed TB or TB/HIV co-morbidity. Data collection started in February 2021 and continued through October 2022. Data were collected through patient surveys and reviews of patients’ health records. Of note, recruitment of participants started during the COVID-19 pandemic and data continued to be collected after the full-scaled invasion of Ukraine.

### Study population and recruitment

The sample for this analysis consisted of 209 patients (100 PLWTB and 109 PLWTBHIV). We recruited adult patients (≥18 years) who had initiated outpatient TB treatment within the past 30 days. We enrolled patients in the study if they had initiated outpatient TB treatment following diagnosis (intensive phase) or if they had recently transitioned from inpatient to outpatient TB treatment (continuous phase). All consecutive patients who fit study criteria and provided informed consent were enrolled in the study.

### Ethics approval and consent to participate

Ethics approval was obtained from the UIPHP Institutional Review Board. All participants were engaged in the informed consent process. Confidentiality was ensured for the study participants by not including names and contact information in the final dataset. The electronic database was password protected and could be accessed only by the principal investigators and co-investigators.

### Data collection

Medical providers at the study sites identified, informed, and referred eligible patients to research assistants. Research assistants informed participants about the study, requested informed consent, and conducted the interviews. Survey data were collected using interviewer-administered questionnaires. Physicians extracted patients’ clinical data related to TB and HIV from their medical charts into a pre-defined form in Microsoft Excel. Survey questionnaires, screening forms, and informed consent were collected via the Research Electronic Data Capture (REDCap) platform [15,16] hosted on the UIPHP server. UIPHP researchers designed, developed, and maintained electronic data collection forms and implemented procedures for data quality control. Preventive quarantine measures against COVID-19 were followed throughout the study as required. Interviews with study participants were conducted either in-person or remotely via phone or internet-based communication technologies (e.g. Zoom, WhatsApp, etc.).

Demographic and socioeconomic variables included type of residence, age, sex, education, marital status, employment, income level, children in the family, housing stability, and history of imprisonment. Depression was assessed using the Center for Epidemiological Studies-Depression Scale (CES-D, 20-item scale with values ranging from 0 to 60) [17]. Participants with a CES-D score ≥ 16 points were considered at risk for depression, whereas CES-D score < 16 points implied no risk for depression. For those with a positive depression screening, willingness to be treated for depression was assessed with the question: ‘Are you planning to seek medical or therapeutic help regarding your present complaints within the next 3 months?’ Responses were categorized using a 5-point Likert scale from 1 – ‘very unlikely’ to 5 ‘very likely.’ Participants unwilling to receive treatment for depressive symptoms were asked for the reasons.

TB stigma was measured with Van Rie et al.’s TB stigma scale [18] and categorized as very low (0–17), low (18–22), moderate (23–27), and high (28–44). Perceived social support was measured via a modified version of the Duke University-University of North Carolina Functional Support Questionnaire [19]. Illicit drug use items were from the European Monitoring Centre for Drugs and Drug Addiction bio-behavioral survey [20]; a dichotomous variable was created for illicit drug use in a past 30 days.

Clinical data extracted from the medical charts included HIV and TB diagnosis, dates of diagnosis, co-morbidities at TB diagnosis, history of previously treated TB, TB drug resistance, social support during TB treatment, serious adverse events during TB treatment, and for patients with TB/HIV coinfection – date of HIV diagnosis, antiretroviral therapy (ART) regimens, and CD4 cell count and HIV viral load with dates of the diagnostic tests.

### Data analysis

Descriptive analyses included the calculation of proportions for categorical variables and mean and standard deviation (SD) or median and interquartile range (IQR) for continuous variables. Bivariable associations for sociodemographic and clinical covariates and (1) HIV status and (2) risk for depression were estimated with logistic regression. Multivariable logistic regression models were constructed to identify correlates of depression risk. Variables were tested in the multivariable model based on findings from the bivariable analysis (i.e. association was significant at *p* ≤ 0.05) as well as based on theoretical considerations (i.e. age and sex are common correlates of depression). The Hosmer–Lemeshow goodness-of-fit test was used to assess model fit. Missing data were due to the refusals to answer the questions; these responses are reflected in Tables 1 and 2 in the ‘Unknown’ category. The level of significance was set at *p* < 0.05. Data analyses were conducted in R version 4.2.2 (The R Foundation for Statistical Computing).

**Table 1.** Sociodemographic characteristics of 209 patients with TB infection in Kyiv and Odesa, Ukraine, 2021–2022, stratified by HIV infection.

*Characteristic*	Total N = 209	TB n = 100 n (%)	TB/HIV n = 109 n (%)	*p*
**Type of residence**				0.800
Rural	35 (17)	16 (16)	19 (18)	
Urban	174 (83)	84 (84)	90 (82)	
**Sex**				0.700
Female	72 (34)	36 (36)	36 (33)	
Male	137 (66)	64 (64)	73 (67)	
**Age, years**				0.007
Mean (SD)	43 (11)	46 (14)	41 (8)	
Median (IQR)	42 (36, 49)	46 (35, 54)	41 (37, 45)	
Range	19, 84	19, 84	21, 67	
**Marital status**				0.500
Married or partnered	97 (46)	49 (49)	48 (44)	
No partner	112 (54)	51 (51)	61 (56)	
**Education**				0.600
Secondary/vocational school or less	158 (76)	74 (74)	84 (77)	
Incomplete higher/higher	51 (24)	26 (26)	25 (23)	
**Employment**				0.400
Working^a^	131 (63)	60 (60)	71 (65)	
Not working^b^	78 (37)	40 (40)	38 (35)	
**Household monthly income**				0.600
<6000 UAH^c^	89 (46)	47 (48)	42 (44)	
≥6000 UAH	104 (54)	51 (52)	53 (56)	
Unknown	16	2	14	
**Have children under 18 years old**				0.200
No	145 (69)	74 (74)	71 (65)	
Yes	64 (31)	26 (26)	38 (35)	
**Homeless**				0.100
No	190 (91)	88 (88)	102 (94)	
Yes	18 (9)	12 (12)	6 (6)	
Unknown	1	0	1	
**History of incarceration**				0.016
No	161 (78)	85 (85)	76 (71)	
Yes	46 (22)	15 (15)	31 (29)	
Unknown	2	0	2	

^a^Full or part time job. ^b^Unemployed, disabled, homemaker, retired, or student. ^c^Equivalent to ~$198–228 USD across the study period.

**Table 2.** Clinical characteristics of 209 patients with TB infection in Kyiv and Odesa, Ukraine, 2021–2022, stratified by HIV infection.

*Characteristic*	Total, N = 209	TB n = 100 n (%)	TB/HIV n = 109 n (%)	*p*
**TB diagnosis (from the beginning of study)**				0.069
Less than 1 month (=<30 days)	176 (84)	89 (89)	87 (80)	
More than 1 month (>30 days)	33 (16)	11 (11)	22 (20)	
**TB treatment during intensive phase**				0.003
Inpatient	16 (8)	2 (2)	14 (13)	
Outpatient	192 (92)	98 (98)	94 (87)	
Unknown	1	0	1	
**Comorbidities except HIV infection**				0.006
No	165 (79)	87 (87)	78 (72)	
Yes	44 (21)	13 (13)	31 (28)	
**TB treatment history**				0.400
New	166 (79)	82 (82)	84 (77)	
Previously treated	43 (21)	18 (18)	25 (23)	
**MDR/XDR-TB**				0.200
No	191 (91)	94 (94)	97 (89)	
Yes	18 (9)	6 (6)	12 (11)	
**Social support from NGOs during TB treatment**				0.600
No	11 (5)	6 (6)	5 (5)	
Yes	198 (95)	94 (94)	104 (95)	
**Perceived social support, score**				0.001
Mean (SD)	24 (7)	26 (5)	23 (8)	
Median (IQR)	27 (22, 30)	27 (24, 30)	25 (19, 28)	
Range	0, 30	4, 30	0, 30	
**Serious adverse events**				0.200
No	206 (99)	100 (100)	106 (97)	
Yes	3 (1)	0 (0)	3 (3)	
**Illicit drug use in a past 30 days**				0.023
No	191 (91)	96 (96)	95 (87)	
Yes	18 (9)	4 (4)	14 (13)	
**Depression**				<0.001
At risk for clinical depression (CESD ≥ 16 scores)	58 (28)	16 (16)	42 (39)	
No risk	151 (72)	84 (84)	67 (61)	
**HIV diagnosis (from the beginning of study)**				
Less than 1 month			33 (30)	
From 1 month to 12 months			27 (25)	
More than a year			49 (45)	
**Initiated ART therapy**				
Yes			107 (98)	
No			2 (2)	
**CD4 cells at TB diagnosis**				
≥200 cells/m3 blood			37 (34)	
<200 cells/m3 blood			72 (66)	
**Viral load at TB diagnosis**				
No viral suppression			80 (77)	
Viral suppression (<200)			24 (23)	
Unknown			5	

## Results

Table 1 presents the socioeconomic and demographic characteristics of the sample, stratified by HIV status. The mean age of participants was 43 years (SD = 11), the median age was 42 (IQR = 36, 49). The sample was majority male 66% (*n* = 137) and lived in urban areas 83% (*n* = 174). Almost half 46% (*n* = 97) were married or had a partner and 24% (*n* = 51) had equivalent to college or higher education (incomplete Bachelor's degree or higher). Approximately 63% (*n* = 131) were employed and 46% (*n* = 89) had a monthly household income below Ukraine’s minimum salary, equivalent to approximately $198–228 USD across the study period [21]. The majority 91% (*n* = 190) of participants were stably housed (i.e. not homeless). Almost one-fourth of participants (22%, *n* = 46) had a history of incarceration. PLWTBHIV were significantly younger and more likely to have a history of incarceration than PLWTB.

Table 2 presents the clinical and behavioral characteristics of the sample, stratified by HIV status. One-fifth of the patients (21%, *n* = 43) had a previously treated TB infection. Eighteen (9%) had MDR-TB or extensively drug-resistant tuberculosis (XDR-TB). Most of the patients with MDR-TB or XDR-TB (67%, *n* = 12) were coinfected with HIV and 39% (*n* = 7) had depression risk. High TB stigma was significantly more common among people with MDR- or XDR-TB compared to those who did not have resistant TB (50% vs. 18%, *p* = 0.021; data not shown), while very low and low TB stigma were less common among those with MDR- or XDR-TB (11% vs. 27% for both categories). PLWTBHIV were significantly more likely to receive inpatient TB treatment (13% [*n* = 14] vs. 2% [*n* = 2], *p* = 0.003) and were more likely to have a comorbidity compared to PLWTB (28% [*n* = 31] vs. 13% [*n* = 13], *p* = 0.006). Besides HIV coinfection, a variety of comorbidities were reported among the sample including hepatitis 10% (*n* = 20), anemia 9% (*n* = 19), hypertension 2% (*n* = 5), candidiasis 2% (*n* = 5), mycobacteriosis 1% (*n* = 3), cancer 1% (*n* = 2), diabetes 1% (*n* = 2), and osteochondrosis 1% (*n* = 2). Less than 1% (*n* = 1) reported each of the following: coronary heart disease, cytomegalovirus infection, peptic ulcer, and depression. Some participants had more than one comorbidity. Only 1% (*n* = 3) experienced serious adverse events during TB treatment. Illicit drug use within past 30 days was reported by 9% (*n* = 18); PLWTBHIV were more likely to report illicit drug use as compared to those with TB infection only (13% [*n* = 14] vs. 4% [*n* = 4], *p* = 0.023).

Almost all participants (95%, *n* = 198) received social support from non-governmental organizations during TB treatment, as indicated in the medical records. Perceived social support among participants was high with the median score of 27 (IQR = 22, 30). More than half of PLWTBHIV were diagnosed with HIV within the year prior to study enrollment 55% (*n* = 60). Almost all PLWTBHIV (98%, *n* = 107) initiated ART for HIV infection. Two-thirds of PLWTBHIV had CD4 cells counts less than 200 cells/mm^3^ (66%, *n* = 72) and 77% (*n* = 80) had detectable viral loads (i.e. equal or more than 200 copies/ml); Table 2).

Approximately 28% (*n* = 58) of the participants were at risk for depression based on a CES-D cut-off score (Table 2). Females were more likely to be at risk for depression as compared to males (35% [*n* = 25] vs. 24% [*n* = 33]); however, this difference was not significant (*p* = 0.10; data not shown). There were also no significant differences in mean (41 [SD = 10] vs. 44 years [SD = 12], t-test *p* = 0.148) and median age by risk of depression (40 vs. 43 years, ranksum *p* = 0.136). Depression risk was more common among PLWTBHIV compared to PLWTB (39% [*n* = 42] vs. 16% [*n* = 16], *p* < 0.001). Among those at risk for depression, 66% (*n* = 37) were willing to seek either therapeutic or medical help for it (data not shown). For the 34% (*n* = 19) who were not willing to seek help, reasons for not seeking help included: not very serious complaints 63% (*n* = 12), unwillingness to take more pills 21% (*n* = 4), not affordable depression treatment 16% (*n* = 3), lack of awareness of where to apply for help 10% (*n* = 2), lack of time to visit a doctor 10% (*n* = 2), no regular complaints 10% (*n* = 2), possible side effects of treatment 5% (*n* = 1), embarrassed to apply to the doctor 5% (*n* = 1), and other reasons 21% (*n* = 4). Among the written in explanations, participants reported a low level of confidence in doctors, symptoms probably related to TB, and no sense in seeking treatment. Some participants reported more than one reason for not seeking help. Both patients who reported pill load as the reason for not seeking for help for depressive symptoms were on ART in addition to TB treatment.

### Multivariable models

In bivariate analyses (Table 3), HIV coinfection, time since TB diagnosis, illicit drug use in a past 30 days, TB stigma, perceived social support, and employment status were significantly associated with the odds of depressive symptoms. These variables, along with sex and age, were included in the full model for multivariable analysis.

**Table 3.** Bivariate analysis and multivariable logistic regression models of depression among 209 patients with TB infection in Kyiv and Odesa, Ukraine, 2021–2022.

Characteristic	Depression (CESD ≥ 16 scores)		Crude OR (95% CI)	Full model Adjusted OR (95% CI)	Parsimonious model Adjusted OR (95% CI)
	Yes n = 58 n (%)	No n = 151 n (%)			
**Group**					
TB	16 (28)	84 (56)	1.0	1.0	1.0
TB/HIV	42 (72)	67 (44)	3.29 (1.73, 6.51)	2.32 (1.09, 5.08)	2.95 (1.46, 6.20)
**Sex**					
Female	25 (43)	47 (31)	1.0	1.0	-
Male	33 (57)	104 (69)	0.60 (0.32, 1.12)	0.49 (0.23, 1.06)	-
**Age, years, mean (SD)**	41 (10)	44 (12)	0.98 (0.95, 1.01)	0.97 (0.93, 1.00)	-
**Employed**					
Working^a^	30 (52)	101 (67)	1.0	1.0	1.0
Not working^b^	28 (48)	50 (33)	1.89 (1.02, 3.50)	2.11 (0.98, 4.61)	2.25 (1.12, 4.60)
**Time since TB diagnosis**					
Less than 30 days	54 (93)	122 (81)	1.0	1.0	-
More than 30 days	4 (7)	29 (19)	0.31 (0.09, 0.84)	0.36 (0.09, 1.12)	-
**Illicit drug use in a past 30 days**					
No	47 (81)	144 (95)	1.0	1.0	1.0
Yes	11 (19)	7 (5)	4.81 (1.79, 13.8)	3.98 (1.23, 13.9)	3.57 (1.18, 11.6)
**TB stigma score**					
Very low (0–17 scores)	3 (5)	51 (34)	1.0	1.0	1.0
Low (18–22 scores)	15 (26)	38 (25)	6.71 (2.04, 30.5)	5.20 (1.42, 25.4)	6.32 (1.81, 29.9)
Moderate (23–27 scores) High (28–44 scores)	19 (33) 21 (36)	39 (26) 23 (15)	8.28 (2.59, 37.1) 15.5 (4.77, 70.5)	7.80 (2.19, 37.9) 11.8 (3.07, 60.6)	7.40 (2.22, 34.1) 15.5 (4.52, 73.2)
**Perceived social support score, median (IQR)**	24 (14, 28)	27 (24, 30)	0.92 (0.88, 0.96)	0.96 (0.91, 1.01)	-

OR = Odds ratio; CI = Confidence interval. ^a^Full or part time job. ^b^Unemployed, disabled, homemaker, retired, or student.

Full and parsimonious logistic models were constructed to estimate the associations between key covariates and risk for depression. In the final parsimonious model (Table 3), HIV coinfection (adjusted odds ratio [AOR] = 2.95, 95% confidence interval [CI]: 1.46–6.20), illicit drug use in a past 30 days (AOR = 3.57, CI: 1.18–11.60), TB stigma (low stigma: AOR = 6.32, CI: 1.81–29.9, moderate stigma: AOR = 7.40, CI: 2.22–34.1, high stigma: AOR = 15.5, CI: 4.52–73.2), and unemployment status (AOR = 2.25, CI: 1.12–4.60) were significantly associated with the risk for depression among people with TB. The Hosmer–Lemeshow goodness-of-fit test for the parsimonious model was non-significant (*p* = 0.12), indicating that the model fit the data well.

## Discussion

The proportion of PLWTB at risk for depression in this sample was higher than general adult population estimates for adults before the full-scale invasion of Ukraine (28% vs. 6%, respectively) [7,22], yet lower than what has been observed among PLWTB elsewhere. According to a 2020 meta-analysis that included 25 studies across low- and middle-income countries, the pooled prevalence of depression among TB patients was 45.2% (95% CI 38.04–52.55) [14], which was considerably higher than the present study. Of note, the meta-analysis did not contain data from any countries in the Eastern European or Central Asian region [23]. The difference in the prevalence of the risk for depression in the present versus prior studies could be explained by differences in the instruments used to collect data, cut-off scores applied to estimate prevalence, populations studied, type of TB (e.g. MDR-TB vs. non-MDR-TB), or sample size. Despite these differences, the findings suggest that PLWTB in Ukraine experience a substantial burden of depression symptomology.

This study highlights the potential for depression screening to identify patients in need of treatment in Ukraine. The patients who screened positive for depression in this study did not have a diagnosis of depression in their medical charts, suggesting missed opportunities for screening and referral to treatment. Only one patient had depression recorded in the medical chart at baseline; this person had a low CESD score suggesting that the depression may have been treated or had resolved at the time of the study. These findings were consistent with the WHO survey on the main risk factors for noncommunicable diseases (NCDs), which reported that only one in four adults with suspected depression were told about it by a doctor or healthcare professional [7].

In this sample, PLWTBHIV had approximately three times the odds of depression compared to PLWTB after controlling for employment status, past 30-day illicit drug use, and TB stigma. This is consistent with prior studies; for example, two studies in Ethiopia found co-morbid HIV infection to increase the odds of depression four to six-fold [24,25]. One study of PLWTB in South Africa found high rates of psychiatric disorders, but no differences in prevalence by HIV status [26]; they did not assess depression by HIV status specifically but did find high prevalence of major depressive episodes (40.6%), unipolar depression (37.6%), and bipolar spectrum conditions (19.3%). Depression may be elevated among PLWHIV coinfection because of HIV stigma [26–28]. Furthermore, psychiatric symptoms, including depression, could occur as a side effect of specific antiretroviral therapies for HIV [29]. In this study, some patients’ ART regimens included dolutegravir and efavirenz, medications associated with increased risk for depression [30,31].

Unemployment was significantly associated with depression. This is consistent with prior studies that have shown unemployment to be associated with depression and anxiety, including among PLWTB [32,33]. Remarkably, in the present study, around half of participants with depression were unemployed (48%), and one-fifth (21%) had permanent or temporary disability.

TB stigma was also a significant correlate of depression. TB stigma has previously been shown to be associated with depression [24,34–36] as well as a barrier to positive TB treatment outcomes [37]. Educational programs aimed at supporting specialized healthcare providers to identify and address patients’ concerns about TB stigma could mitigate stigma impact. Support from the family and communities could also alleviate stigma [37].

Approximately two-thirds of those who screened positive for depression risk were willing to seek medical or therapeutic help. Among participants unwilling to seek help, the reasons for not doing so were consistent with the barriers to seeking care reported in the Mental Health Assessment for Ukraine [22]. Of note, the full-scale invasion of Ukraine is likely to exacerbate mental health issues, including depression and other mood disorders in this population. Recent evidence has demonstrated an increase in depression, anxiety, and stress among Ukrainians [38].

This study has several limitations to consider. The study population is limited to TB patients in Odesa and Kyiv and therefore is not representative of all PLWTB in Ukraine. The study was initiated during the second year of the COVID-19 pandemic and the last 5 months of baseline enrollment coincided with Russia’s invasion of Ukraine. The COVID-19 pandemic and the full-scale invasion of Ukraine did not affect the willingness of participants to join the study (interviews were remote). However, recruitment of newly diagnosed patients, the target group, was likely negatively affected as people may have delayed treatment due to the pandemic or military conflict, or may have been displaced to other parts of Ukraine, or out of Ukraine. Therefore, the primary study extended the sample by recruiting TB patients without HIV comorbidity (not only with TB/HIV coinfection as initially was planned). It is also plausible that mental health was negatively affected by both the pandemic and the invasion. Due to the limited sample size, confidence intervals are wide for some associations and estimates should be interpreted with caution.

## Conclusions

The WHO recommends integrating screening for NCDs, including mental health conditions, into infectious disease programs [39]. In 2023, the Ukrainian government stipulated that screening should be provided to patients with sensitive and multidrug-resistant TB with the PHQ-9 and Hospital Anxiety and Depression Scale (HADS) [40]. Similar provisions were made by the government for PLHIV, where the PHQ-2 should be used to screen for depression and anxiety and those with a positive answer to at least one of the PHQ-2 questions being further evaluated with the PHQ-9 [41]. These results suggest that Ukrainian patients with TB and/or HIV would benefit from the integration of brief mental health screenings and referrals to treatment in tuberculosis care, whether it is provided in primary, secondary, or tertiary care settings and regardless of whether the TB infection is MDR or XDR. Additional research is needed to determine how to effectively integrate and implement such tools into these settings.

Linkage of clinical care for TB/HIV with mental health services should continue to be established, in light of the substantial depression risk among this population. For PLWTB and comorbidities such as HIV, depression, and substance use in the context of challenging social determinants of health such as stigma and unemployment, accessibility of comprehensive services is critical for supporting their health and well-being.
